# Prognostic Landscape of Lymphoepithelial Carcinoma: Analysis of a National Database

**DOI:** 10.7759/cureus.78828

**Published:** 2025-02-10

**Authors:** Beau Hsia, Yanick Tade, Susan Rafie, Shervin Harirchian, Gejla Toromani, Daniela Hailyn Gonzalez Alejandro, Peter T Silberstein

**Affiliations:** 1 Otolaryngology—Head and Neck Surgery, Creighton University School of Medicine, Phoenix, USA; 2 Orthopedic Surgery, Creighton University School of Medicine, Omaha, USA; 3 Medicine, Midwestern University Arizona College of Osteopathic Medicine, Glendale, USA; 4 Medicine, Creighton University School of Medicine, Phoenix, USA; 5 Biology, Miami University, Oxford, USA; 6 Chemical Engineering, Universidad Juárez Autónoma de Tabasco, Tabasco, MEX; 7 Oncology, Creighton University School of Medicine, Omaha, USA

**Keywords:** adjuvant radiation, lymphoepithelial carcinoma, national cancer database, ncdb, overall survival, socioeconomic factors

## Abstract

Objective: Lymphoepithelial carcinoma (LEC) is a rare and malignant epithelial tumor characterized by poorly differentiated carcinoma with lymphoplasmacytic infiltrate. This study utilizes the National Cancer Database (NCDB) to analyze demographic, clinical, and treatment factors affecting overall survival and evaluate the impact of treatment modalities and socioeconomic factors on outcomes.

Methods: Patients with LEC were identified in the NCDB between 2004 and 2020. Kaplan-Meier survival analysis, log-rank tests, and multivariable Cox proportional hazard models were used to assess the impact of variables such as age, tumor stage, Charlson-Deyo comorbidity score, adjuvant therapies, and socioeconomic factors.

Results: This study of 408 LEC patients identified several significant predictors of mortality. Advanced cancer stage (hazard ratio (HR): 8.45; 95% confidence interval (CI): 4.25-16.79; p < 0.001) and increasing age (per five years; HR: 1.20; 95% CI: 1.12-1.28; p < 0.001) were associated with increased mortality risk. Patients with lung LEC had worse survival than those with LEC in the female reproductive organs (HR: 0.35; 95% CI: 0.20-0.64; p < 0.001), while those with head and neck LEC had better survival rates than those with lung LEC (HR: 1.66; 95% CI: 1.04-2.67; p = 0.036). Chemotherapy was associated with improved survival (HR: 0.53; 95% CI: 0.30-0.96; p = 0.037). Socioeconomic status also played a role, with residence in the lowest educational attainment quartile associated with poorer survival (HR: 0.51; 95% CI: 0.26-0.98; p = 0.044) and private insurance coverage associated with better survival (HR: 1.97; 95% CI: 1.02-3.80; p = 0.042).

Conclusion: Older age, advanced stage, and low educational attainment are key predictors of poorer survival in LEC. Lymphoepithelial carcinoma has the worst prognosis when found in the lungs compared to any other primary site. Primary chemotherapy significantly improves survival, emphasizing its role in treatment. Further research is needed to explore cancer-specific mortality and the impact of site-specific differences.

## Introduction

Lymphoepithelial carcinoma (LEC) is a rare epithelial malignancy. The WHO classification defines LEC as a poorly differentiated squamous cell carcinoma or an undifferentiated carcinoma exhibiting a prominent lymphoplasmacytic infiltrate, sharing morphological similarities with nasopharyngeal carcinoma [1-6]. It differs from nasopharyngeal carcinoma (NPC) based on location and clinical outcome.

Lymphoepithelial carcinoma has been identified in a variety of anatomical sites, including the upper and lower lobes of the lung, uterine cervix, thymus, oral cavity, oropharynx, nasal cavity, paranasal sinuses, and other head and neck locations, with the major salivary glands being the most commonly affected [1]. Data from the Surveillance, Epidemiology, and End Results (SEER) program suggest that LEC accounts for approximately 5% of all head and neck malignancies [7]. Epstein-Barr virus (EBV) association is particularly strong in LEC arising in the oral cavity, oropharynx, nasal cavity, and paranasal sinuses in endemic regions such as Southeast Asia [1,8-11]. However, the existing literature primarily consists of small case reports and case series from these endemic areas. Studies in non-endemic regions are limited, reflecting the rarity of LEC in these populations [7,12].

The age at the time of diagnosis for LEC varies considerably, with a peak incidence between 50 and 70 years of age [3,7]. The gender distribution varies by anatomical site, with some studies reporting male predominance in head and neck LEC but female predominance in oral and oropharyngeal cases [13-16]. While smoking and alcohol consumption are recognized risk factors for many head and neck cancers, their specific association with LEC has not been well established in the literature due to the rarity of this malignancy [3,7,13-16]. This malignancy has a five-year overall survival rate of 57% to 90%, irrespective of histological grade [9,17,18]. Due to the rarity of LEC, limited data exist regarding factors influencing overall survival. Prior studies have suggested that older age, advanced tumor stage, higher histological grade, larger tumor size, and the absence of surgical resection are associated with poorer outcomes [19]. Standard treatment typically involves surgical excision followed by postoperative radiotherapy (PORT) as adjuvant therapy. However, the efficacy of PORT and the optimal criteria for its administration remains uncertain [12].

The primary objective of this study was to identify independent prognostic factors affecting overall survival in patients with LEC. Secondary objectives included: 1) evaluating the impact of different treatment modalities (surgery, radiation, and chemotherapy) on survival; 2) assessing the relationship between socioeconomic factors (income, education, and insurance status) and outcomes; 3) analyzing site-specific survival differences across anatomical locations; and 4) determining the influence of demographic and clinical characteristics on prognosis. This study aims to address the critical gap in knowledge regarding prognostic factors in LEC by analyzing the largest US cohort to date.

## Materials and methods

This is a retrospective cohort study of patients within the National Cancer Database (NCDB) diagnosed with LEC from 2004 to 2020. The NCDB is a clinical oncology database derived from hospital registry data with information on patient characteristics, tumor staging, tumor histology, type of first treatment, disease recurrence, and survival from more than 1,500 Commission on Cancer-accredited facilities. This de-identified patient data was made accessible to the authors through the Participant User Data Files program. 

Patients with LEC were identified from NCDB data using the International Classification of Diseases for Oncology-3rd edition (ICD-O-3) histology code 8082, which classifies tumors based on their morphological and histological features. Patients were selected if they had behavior code 3 (invasive). Patients were excluded from the cohort if they had any concurrent tumors or any missing clinical or demographic data.

Covariates

Patient data were analyzed according to age, sex, race, education level, income, insurance status, tumor size, analytical stage, Charlson-Deyo (CD) comorbidity score, primary anatomic site, primary radiation therapy, primary and adjuvant chemotherapy, and distance traveled for healthcare. Race was categorized into three groups: White, African American, and Other. The 'Other' race category included American Indian/Alaska Native, Asian (including Chinese, Japanese, Filipino, Hawaiian, Korean, Vietnamese, Kampuchean, Asian Indian/Pakistani, Micronesian, and other/unspecified Asian groups), and Pacific Islander (including Native Hawaiian and other/unspecified Pacific Islander groups). Ethnicity was separately categorized as Hispanic or non-Hispanic, following NCDB classification standards. Income was represented by median household income (2016 to 2020) based on the patient's residential zip code at the time of diagnosis. Education level was defined as the percentage of residents within the patient's residential zip code (2020 data) who did not complete high school. The tumor stage (I-IV) was determined using the NCDB analytical stage, prioritizing the pathologic stage and using the clinical stage if pathologic staging was unavailable. Insurance status was classified into five categories: uninsured, private insurance, Medicare, Medicaid, and other government insurance. The primary anatomic site was categorized into four regions based on ICD-O-3 topography codes: head and neck, lung, female reproductive, and other soft tissues. Distance traveled for healthcare was calculated as the mileage between the patient's residence and the reporting hospital. The CD comorbidity score was used to stratify patients into comorbidity groups of 0, 1, 2, and ≥3.

The primary outcome was overall survival (OS), defined as the time from diagnosis to death, with censoring at the date of last contact. Independent prognostic factors were identified using multivariable Cox proportional hazards regression. Kaplan-Meier curves were generated, and OS was estimated at two, five, and 10 years using life tables. The multivariable Cox model included the following a priori variables: age, sex, race, insurance status, education level, income, Charlson-Deyo (CD) comorbidity score, analytical stage, primary anatomic site, surgery of primary site, primary/adjuvant radiation therapy, and primary/adjuvant chemotherapy. A robust sandwich covariance matrix was employed to account for the clustering of patients within facilities. The proportional hazards assumption for each variable was evaluated using log-negative-log survival curves and tests for statistical interaction with time.

Statistical considerations

Cox proportional hazards regression was selected to model survival data due to its ability to handle censored observations and evaluate multiple prognostic factors simultaneously. Kaplan-Meier curves were generated to visualize unadjusted survival differences, while life tables provided standardized survival estimates at clinically actionable time points (two, five, and 10 years). These methods align with recommendations for cancer registry analyses by the NCDB and the American Joint Committee on Cancer (AJCC). The descriptive statistics, unadjusted survival analysis, and multivariable analysis for this study were conducted using SPSS Statistics version 27 (IBM Corp., Armonk, NY, USA). Patients with any missing clinical or demographic factors were excluded from the cohort. Bonferroni correction was used to adjust the p-value threshold for multiple comparisons to p < 0.0083 (0.05/6), ensuring the family-wise error rate remained controlled at α = 0.05. This conservative approach was preferred over false discovery rate control given the exploratory nature of socioeconomic factor analyses. Multicollinearity among socioeconomic variables (income, education, and insurance status) was assessed using variance inflation factors (VIF) derived from linear regression models. All VIFs were <2.0, well below the threshold of 5.0, indicating no significant collinearity.

Oversight

The University of Arizona Biomedical Institutional Review Board (IRB) reviewed this study (IRB submission ID: STUDY00003534) and determined that it does not involve human subjects research as defined by the Department of Health and Human Services (DHHS) and Food and Drug Administration (FDA) regulations. Consequently, IRB approval and ongoing review were not required.

## Results

The study cohort comprised 408 patients diagnosed with LEC. The cohort was predominantly female (n = 258; 59.9%) and White (n = 288; 66.8%). The median age at diagnosis was 60 years. Patients were stratified by income, with 152 (35.4%) residing in areas with a median household income of ≥$74,063. The majority of patients were non-Hispanic (n = 342; 83.8%), while 66 (16.2%) identified as Hispanic or Latino patients. Approximately 131 (30.4%) patients resided in zip codes where >15.3% of residents lacked a high school diploma, whereas 70 (16.2%) resided in zip codes where <5% lacked such education. A total of 191 (44.3%) cases were treated at academic/research institutions. More patients presented with a lower disease stage, with 209 (48.5%) patients presenting with stage I disease and 59 patients (13.7%) presenting with stage IV disease.

All patients presented with invasive tumors. The cohort exhibited a low comorbidity burden, with 67.9% (n = 292) having a CD comorbidity score of 0. Most treatments (n = 181; 44.3%) were administered at academic or research institutions, with 21.8% (n = 89) of patients receiving treatment in the South Atlantic region. The majority of patients (n = 250; 61.3%) resided in metropolitan areas with populations exceeding 1 million. In terms of insurance coverage, 44.3% (n = 191) had private or managed care insurance, followed by Medicare (n = 161; 37.4%) and Medicaid (n = 61; 14.2%). Surgical resection of the tumor was the most frequently utilized treatment (n = 305; 70.8%). Postoperative assessment revealed no residual tumor in the majority of patients (n = 287; 66.6%). Radiation therapy was the primary treatment modality for 40.0% (n = 163) of patients. In terms of adjuvant therapies, 79 (18.3%) patients received adjuvant radiation therapy, and 88 (20.4%) received adjuvant chemotherapy. Descriptive statistics are presented in Tables 1-3.

**Table 1:** Clinical and demographic characteristics of 431 patients with LEC LEC: Lymphoepithelial carcinoma, CD: Charlson-Deyo

Variable		N (%)
Sex	Male	173 (40.1%)
	Female	258 (59.9%)
Race	White	288 (66.8%)
	African American	76 (17.6%)
	Other	67 (15.5%)
Ethnicity	Hispanic	66 (16.2%)
	Non-Hispanic	342 (83.8%)
Age (years)	Mean ± standard deviation	56.31 ± 120.47
	Median (interquartile range)	60.00 (83)
Zip code-level median household income (2016-2020, $)	< $46,277	88 (20.0%)
	$46,277-$57,856	76 (17.6%)
	$57,857-$74,062	115 (26.7%)
	≥ $74,063	152 (35.3%)
Zip code-level education (% without high-school degree, 2020)	≥ 15.3%	131 (30.4%)
	9.1%-15.2%	119 (27.6%)
	5%-9%	111 (25.8%)
	< 5%	70 (16.2%)
Insurance status	Uninsured	15 (3.5%)
	Private	191 (44.3%)
	Medicaid	61 (14.2%)
	Medicare	161 (37.4%)
	Other government insurance plans	3 (0.7%)
Distance traveled for health care (miles)	Mean ± Standard deviation	22.61 ± 39.44
	Median (interquartile range)	10.70 (20.6)
CD comorbidity score	0	292 (67.7%)
	1	101 (23.4%)
	2	22 (5.1%)
	≥ 3	16 (3.7%)

**Table 2:** Tumor characteristics of 431 patients with LEC LEC: Lymphoepithelial carcinoma

Variable	N (%)
Stage I	209 (48.5%)
Stage II	71 (16.5%)
Stage III	92 (21.3%)
Stage IV	59 (13.7%)

**Table 3:** Treatment characteristics of 431 patients with LEC LEC: Lymphoepithelial carcinoma, NOS: Not otherwise specified

Variable		N (%)
Treatment	Surgery	305 (70.8%)
	Primary radiation	167 (38.7%)
	Primary chemotherapy	192 (44.5%)
	Adjuvant chemotherapy	88 (20.4%)
	Adjuvant radiation	79 (18.3%)
Surgical margins	No residual tumor	287 (66.6%)
	Residual tumor, NOS	11 (2.6%)
	Microscopic residual tumor	18 (4.2%)
	Macroscopic residual tumor	3 (0.7%)

On multivariate analysis, each additional five years of age was associated with a 20% increase in mortality risk (hazard ratio (HR): 1.20; 95% confidence interval (CI) 1.12-1.28; p < 0.001). The advanced stage was a significant predictor of mortality. Compared with stage I, patients with stage II disease had a 156% greater chance of mortality (HR: 2.56; 95% CI: 1.47-4.45; p < 0.001), those with stage III disease had a 256% greater chance (HR: 3.56; 95% CI: 2.10-6.05; p < 0.001), and those with stage IV disease had a 745% greater chance (HR: 8.45; 95% CI: 4.25-16.79; p < 0.001). White race was associated with a significantly increased risk of mortality compared to other races (HR: 0.38; 95% CI: 0.20-0.71; p = 0.003). Surprisingly, patients residing in zip codes with the lowest median household incomes (<$46,277) experienced a significantly higher risk of mortality compared to those with median household incomes between $57,857 and $74,062 (HR: 1.96; 95% CI: 1.07-3.58; p = 0.030). Compared to patients residing in zip codes where <5.0% of residents lacked a high school diploma, patients residing in areas where ≥15.3% of residents lacked a high school diploma demonstrated a significantly higher risk of mortality (HR: 0.51; 95% CI: 0.26-0.98; p = 0.044). Similarly, compared to patients residing in zip codes where <5.0% of residents lacked a high school diploma, patients residing in areas where 9.1% to 15.2% of residents lacked a high school diploma also demonstrated a significantly increased risk of mortality (HR: 0.51; 95% CI: 0.29-0.90; p = 0.020). Patients with private insurance had a significantly decreased risk of mortality compared to those with Medicaid (HR: 1.97; 95% CI: 1.02-3.80; p = 0.042).

The primary site of LEC had a notable impact on survival. Specifically, patients with LEC in the lung experienced significantly worse survival compared to those with LEC in the female reproductive organs (HR: 0.35; 95% CI: 0.20-0.64; p < 0.001). Similarly, patients with head and neck LEC demonstrated significantly better survival compared to those with lung LEC (HR: 1.66; 95% CI: 1.04-2.67; p = 0.036). Patients with LEC in other soft tissues showed significantly better survival compared to those in female reproductive organs (HR: 2.83; 95% CI: 1.56-5.13; p < 0.001), but no statistically significant difference in survival when comparing LEC in the head and neck or the lung to LEC in other soft tissues.

Higher comorbidity (CD score ≥3) was not significantly associated with increased mortality compared to a score of 0 (HR: 1.33; 95% CI: 0.53-3.32; p = 0.543). Kaplan-Meier survival analysis showed that patients receiving adjuvant radiation therapy had numerically higher estimated five-year survival probabilities of 79.8% (95% CI: 56.8%-100.0%) and 10-year survival probabilities of 63.6% (95% CI: 24.8%-100.0%), compared with those who did not receive adjuvant therapy, which had five-year survival probabilities of 70.4% (95% CI: 47.4% to 93.4%) and 10-year survival probabilities of 53.1% (95% CI: 33.5% to 72.7%). However, this difference did not reach statistical significance (p = 0.093). Similarly, the multivariate Cox regression analysis showed no statistically significant differences in survival between patients who did and did not receive surgery (p = 0.210), primary radiation (p = 0.435), or adjuvant radiation (p = 0.415). However, the use of primary chemotherapy was associated with a statistically significant increase in survival (HR: 0.53; 95% CI: 0.30-0.96; p = 0.037). Survival and prognostic data are presented in Tables 4-5.

**Table 4:** Median survival times in LEC (univariate analysis) LEC: Lymphoepithelial carcinoma, NCDB: National Cancer Database

Variable		Two-year% (95% CI)	Five-year% (95% CI)	10-year% (95% CI)
Sex (p = 0.660)	Male	89.8 (85.3-94.3)	71.8 (65.1-78.5)	49.6 (42.1-57.1)
	Female	84.2 (79.7-88.7)	72.4 (66.9-77.9)	58.9 (52.9-64.9)
Race (p < 0.001)	White	82.0 (77.6-86.4)	66.4 (60.9-71.9)	46.9 (41.1-52.7)
	Black	95.7 (91.1-100.3)	87.5 (80.1-94.9)	76.8 (67.3-86.3)
	Other	95.0 (89.8-100.2)	80.7 (71.2-90.2)	73.2 (62.6-83.8)
Zip code-level median household income (p = 0.013)	< $46,277	88.2 (81.5-94.9)	84.2 (76.6-91.8)	67.5 (57.7-77.3)
	$46,277-$57,856	85.4 (77.5-93.3)	68.3 (57.8-78.8)	52.3 (41.1-63.5)
	$57,857-$74,062	81.6 (74.5-88.7)	64.6 (55.9-73.3)	46.4 (37.3-55.5)
	≥ $74,063	89.4 (84.5-94.3)	72.5 (65.4-79.6)	55.8 (47.9-63.7)
Zip code-level education (% without high-school degree, p = 0.126)	≥ 15.3%	85.7 (79.7-91.7)	76.6 (69.3-83.9)	63.3 (55.0-71.6)
	9.1%-15.2%	87.3 (81.3-93.3)	71.3 (63.2-79.4)	49.8 (40.8-58.8)
	5%-9%	86.4 (80.0-92.8)	69.2 (60.6-77.8)	48.8 (39.5-58.1)
	< 5%	86.4 (78.4-94.4)	70.1 (59.4-80.8)	61.7 (50.3-73.1)
Age (years, p < 0.001)	0-25	93.7 (88.0-99.4)	93.7 (88.0-99.4)	83.6 (74.9-92.3)
	26-50	93.2 (88.3-98.1)	85.4 (78.5-92.3)	77.3 (69.1-85.5)
	51-75	85.9 (80.5-91.3)	68.5 (61.3-75.7)	53.0 (45.3-60.7)
	76-100	75.0 (66.5-83.5)	53.2 (43.4-63.0)	22.1 (14.0-30.2)
NCDB analytical stage (p < 0.001)	I	89.1 (84.9-93.3)	76.4 (70.6-82.2)	58.4 (51.7-65.1)
	II	92.3 (87.1-97.5)	72.8 (64.1-81.5)	60.6 (51.1-70.1)
	III	83.9 (68.5-99.3)	68.4 (49.0-87.8)	47.9 (27.0-68.8)
	IV	73.0 (51.2-94.8)	62.7 (39.0-86.4)	51.2 (26.7-75.7)

**Table 5:** Multivariable Cox regression model of 431 patients with LEC LEC: Lymphoepithelial carcinoma

Variable		HR (95% CI)	p-values
Age (five years)		1.20 (1.12 - 1.28)	< 0.001
Sex: Males vs. females		1.14 (0.78 - 1.66)	0.491
Race	White vs. Black	0.63 (0.32 - 1.23)	0.176
	White vs. Other	0.38 (0.20 - 0.71)	0.003
	Black vs. Other	0.60 (0.24 - 1.47)	0.260
Charlson-Deyo Score	0 vs. 1	0.96 (0.64 - 1.46)	0.867
	0 vs. 2	1.48 (0.76 - 2.89)	0.251
	0 vs. ≥ 3	1.33 (0.53 - 3.32)	0.543
	1 vs. 2	1.53 (0.76 - 3.10)	0.235
	1 vs. ≥ 3	1.38 (0.54 - 3.54)	0.507
	2 vs. ≥ 3	0.90 (0.32 - 2.52)	0.839
Zip code-level median household income (2020 US Dollars)	< $46,277 vs. $46,227-$57,856	1.52 (0.82 - 2.80)	0.181
	< $46,277 vs. $57,857-$74,062	1.96 (1.07 - 3.58)	0.030
	< $46,277 vs. ≥ $74,063	1.55 (0.80 - 3.00)	0.195
	$46,227-$57,856 vs. $57,857-$74,062	1.29 (0.77 - 2.15)	0.331
	$46,227-$57,856 vs. ≥ $74,063	1.02 (0.59 - 1.75)	0.941
	$57,857-$74,062 vs. ≥ $74,063	0.79 (0.51 - 1.22)	0.293
Zip code-level education (2020, % No high-school diploma)	≥ 15.3% vs. 9.1%-15.2%	1.00 (0.60 - 1.66)	0.988
	≥ 15.3% vs. 5.0%-9.0%	0.69 (0.39 - 1.22)	0.196
	≥ 15.3% vs. < 5.0%	0.51 (0.26 - 0.98)	0.044
	9.1%-15.2% vs. 5.0%-9.0%	0.69 (0.43 - 1.10)	0.120
	9.1%-15.2% vs. < 5.0%	0.51 (0.29 - 0.90)	0.020
	5.0%-9.0% vs. < 5.0%	0.74 (0.42 - 1.30)	0.296
Insurance	None vs. Private	0.72 (0.21 - 2.41)	0.591
	None vs. Medicaid	1.42 (0.38 - 5.21)	0.602
	None vs. Medicare	0.83 (0.24 - 2.92)	0.774
	Private vs. Medicaid	1.97 (1.02 - 3.80)	0.042
	Private vs. Medicare	1.16 (0.71 - 1.88)	0.550
	Medicaid vs. Medicare	0.59 (0.28 - 1.24)	0.163
Treatment	No surgery vs. surgery	0.71 (0.42 - 1.21)	0.210
	No primary chemotherapy vs. primary chemotherapy	0.53 (0.30 - 0.96)	0.037
	No primary radiation vs. primary radiation	1.27 (0.70 - 2.03)	0.435
	No adjuvant chemotherapy vs. adjuvant chemotherapy	0.61 (0.31 - 1.21)	0.154
	No adjuvant radiation vs. adjuvant radiation	0.76 (0.39 - 1.48)	0.415
Primary site	Lung vs. female reproductive organs	0.35 (0.20 - 0.64)	<0.001
	Head and neck vs. lung	1.66 (1.04 - 2.67)	0.036
	Head and neck vs. female reproductive organs	0.59 (0.31 - 1.13)	0.111
	Other vs. head and neck	0.94 (0.25 - 3.46)	0.926
	Other vs. lung	1.70 (0.89 - 3.27)	0.111
	Other vs. female reproductive organs	2.83 (1.56 - 5.13)	<0.001
Stage	Stage I vs. Stage II	2.56 (1.47 - 4.45)	<0.001
	Stage I vs. Stage III	3.56 (2.10 - 6.05)	<0.001
	Stage I vs. Stage IV	8.45 (4.25 - 16.79)	<0.001
	Stage II vs. Stage III	1.39 (0.82 - 2.38)	0.225
	Stage II vs. Stage IV	3.30 (1.68 - 6.48)	<0.001
	Stage III vs. Stage IV	2.37 (1.27 - 4.44)	0.007

## Discussion

This study comprehensively evaluates demographic, clinical, and treatment factors influencing survival in patients with LEC, identifying key prognostic indicators. The cohort comprised 59.9% female patients, which is somewhat consistent with existing literature. A large-scale analysis of the SEER database reported a male predominance in head and neck LEC (69.7% male vs. 30.3% female) [20], while a systematic review of oral and oropharyngeal LEC cases indicated a slight female predominance (57% female vs. 43% male) [1]. The inclusion of female reproductive tract LEC in this study may contribute to the observed female predominance. This variability in sex distribution across different anatomical sites, as highlighted by the contrasting findings in head and neck versus oral/oropharyngeal LEC, suggests the potential influence of site-specific etiological factors. This study noted significant differences in site-specific survival rates, consistent with existing literature highlighting substantial differences in survival depending on the LEC site. Pulmonary LEC demonstrated worse survival than LEC of the female reproductive organs or head and neck, corroborating previous reports of a poorer prognosis for pulmonary LEC [21].

The stage at diagnosis remains a pivotal prognostic factor. The stage distribution in this cohort showed 35% of patients presenting with stage III-IV disease, which appears lower than reports from a Chinese cohort where 66% presented with stage III-IV disease, though the direct statistical comparison was not possible without access to patient-level data [21]. The 8.45-fold increase in mortality risk observed in stage IV patients compared to stage I aligns with prior estimates, reinforcing the poor survival associated with advanced-stage disease [21]. Notably, the magnitude of the effect for advanced-stage disease (HR 8.45) far exceeded that of socioeconomic factors (e.g., uninsured status: HR 1.8), underscoring the primacy of early detection in improving outcomes. However, socioeconomic disparities remained comparable to clinical factors like comorbidity burden (CD score ≥3: HR 1.9), highlighting their clinical relevance and potential for intervention. These findings underscore the critical importance of early detection in improving prognosis [22] and highlight the need for initiatives aimed at improving early detection, particularly in underserved populations, to reduce late-stage presentations.

The study also evaluated the efficacy of multimodal treatment strategies. Surgery was the most commonly utilized treatment modality, consistent with evidence supporting its central role in managing LEC of the head and neck, uterus, and lung [2,9,21]. However, this analysis did not find a survival benefit associated with surgery at the primary site, contrasting with prior studies. This discrepancy may be attributable to the overrepresentation of stage I LEC within the NCDB, which differs from the previous studies reporting the efficacy of surgery in later-stage presentations [2,9,19,21]. The absence of a significant survival benefit for surgery may also reflect confounding by tumor resectability or surgical margins, which were not analyzed in this study. For example, patients with microscopically positive margins (R1 resection) may have worse outcomes that offset the benefits of resection. Additionally, the overrepresentation of stage I disease in our cohort, where watchful waiting may be comparable to surgery, could dilute observed effects. Future studies should incorporate surgical quality metrics to clarify this relationship.

In the present study, primary chemotherapy was the only treatment modality found to significantly improve overall survival. This association between chemotherapy use and increased survival in earlier-stage LEC has also been reported in the literature [21]. While this study found no survival benefit with the use of adjuvant therapies, prior studies have presented conflicting evidence regarding the impact of adjuvant therapies, with some showing no survival benefit (e.g., in the larynx and hypopharynx) and others supporting their use [2,21]. While our findings suggest a survival advantage for primary chemotherapy, this association must be interpreted cautiously. Patients selected for chemotherapy may have had better performance status and earlier-stage disease, factors incompletely captured in the NCDB. Future studies should include prospective trials to validate the therapeutic efficacy of chemotherapy in LEC.

Socioeconomic factors, including income, education, and urban residency, demonstrated correlations with patient outcomes. While a substantial portion of the cohort belonged to the highest income bracket, consistent with prior research linking affluence to better healthcare access and treatment adherence, many patients also resided in areas with significant educational disadvantages [23]. This study found a significant decrease in survival for patients from zip codes with the lowest percentage of high school graduates and those from the lowest income regions, as compared with certain higher quartiles, confirming the positive association between educational attainment and survival outcomes. Patients with private insurance demonstrated superior outcomes compared with those insured by Medicaid. Overall, significant associations were found between survival and socioeconomic indicators such as income, education, and insurance type, corroborating earlier studies identifying socioeconomic status as a key determinant of cancer outcomes [23]. The mechanisms linking socioeconomic disadvantage to poor outcomes likely involve multifactorial barriers, including delayed diagnosis, reduced access to specialized care, and lower adherence to treatment. For instance, uninsured patients may face delays in diagnostic imaging or chemotherapy initiation, exacerbating disease progression. These findings align with disparities observed in other cancers, suggesting systemic inequities rather than biological differences [23]. Further research is needed to fully understand these complex dynamics and inform policies, including interventions like patient navigation programs and Medicaid expansion, aimed at reducing socioeconomic disparities in cancer care.

This study has limitations inherent to its retrospective cohort design, including reliance on pre-existing data collection practices within the NCDB. This limited our ability to capture granular clinical details, such as detailed treatment responses, specific chemotherapy regimens, or the precise timing and sequence of multimodal therapies, which restricts the interpretation of optimal treatment sequencing (e.g., neoadjuvant vs. adjuvant approaches). Additionally, the NCDB's comorbidity data is limited to those captured using the CD index, potentially missing clinically relevant comorbidities that could influence survival. Missing data within the cohort further constrained the analysis, potentially introducing bias if missingness was non-random. While we employed statistical methods to adjust for potential confounders available within the database, residual confounding from unmeasured or unknown factors, including molecular/genetic variables (e.g., Epstein-Barr virus status) or treatment sequence details, cannot be entirely excluded.

Furthermore, the numerically small sample size precluded meaningful analysis of treatment effectiveness stratified by disease stage, which could have provided valuable insights into optimal stage-specific treatment approaches. The rarity of LEC also limits our ability to assess the statistical impact of different surgical approaches or radiation treatment planning on outcomes. The absence of cause-specific mortality data further limits conclusions about treatment efficacy, particularly in older patients where competing risks (e.g., cardiovascular mortality) may confound survival estimates.

Furthermore, the NCDB records only overall survival, precluding differentiation between LEC-specific and all-cause mortality. This limits our ability to definitively attribute deaths to the cancer itself. However, given the rarity of this subtype and the large scale of this study, overall survival remains a relevant and meaningful endpoint for evaluating long-term patient outcomes, particularly in the context of population-based research where cause-of-death data may be less reliable.

Despite the NCDB's comprehensiveness, including over 70% of new cancer diagnoses in the US, it excludes data from non-Commissioned on Cancer (CoC)-accredited facilities. This exclusion could introduce selection bias, as patients treated at CoC-accredited centers may differ in socioeconomic status, access to advanced therapies, or provider expertise compared to non-CoC facilities. The study's strengths include its moderate sample size given the rarity of LEC, providing greater statistical power compared to smaller single-institution studies. The consistency of our findings with existing literature, particularly regarding the prognostic significance of stage at diagnosis and primary site, provides further support for our conclusions and strengthens the generalizability of our findings within the limitations of the database. Finally, the US-only population and lack of international data limit broader generalizability to global healthcare contexts.

## Conclusions

This study provides insights into the prognostic factors influencing survival in patients with LEC. Consistent with prior literature, advanced age and advanced-stage disease were identified as independent predictors of poorer survival, emphasizing the critical need for early detection and timely intervention, particularly in the elderly. Lymphoepithelial carcinoma in the lung showed the worst prognosis. Surgery and adjuvant therapies were not found to significantly increase survival, while primary chemotherapy did. Socioeconomic factors demonstrated a statistically significant association with survival in this analysis, which underscores the need for policy changes that aim to improve access to quality healthcare for all socioeconomic groups, thereby potentially improving outcomes for patients with LEC. Future research should focus on exploring specific indications of treatment and investigating cancer-specific mortality to develop more refined prognostic models and ultimately improve patient outcomes in this rare cancer.
